# The Influence of Urinary Concentrations of Organophosphate Metabolites on the Relationship between BMI and Cardiometabolic Health Risk

**DOI:** 10.1155/2015/687914

**Published:** 2015-08-20

**Authors:** Mahsa Ranjbar, Michael A. Rotondi, Chris I. Ardern, Jennifer L. Kuk

**Affiliations:** School of Kinesiology and Health Science, York University, Toronto, ON, Canada M3J 1P3

## Abstract

The objective was to determine whether detectable levels of OP metabolites influence the relationship between BMI and cardiometabolic health. This cross-sectional study was conducted using 2227 adults from the 1999–2008 NHANES datasets. Urinary concentrations of six dialkyl phosphate metabolites were dichotomized to above and below the detection limit. Weighted multiple regression analysis was performed adjusting for confounding variables. Independent of BMI, individuals with detectable metabolites had higher diastolic blood pressure (for dimethylphosphate, diethylphosphate, and diethyldithiophosphate; *P* < 0.05), lower HDL (for diethyldithiophosphate; *P* = 0.02), and higher triglyceride (for dimethyldithiophosphate; *P* = 0.05) than those below detection. Contrarily, those with detectable dimethylthiophosphate had better LDL, HDL, and total cholesterol, independent of BMI. Individuals at a higher BMI range who had detectable diethylphosphate (interaction: *P* = 0.03) and diethylthiophosphate (interaction: *P* = 0.02) exhibited lower HDL, while little difference existed between OP metabolite detection statuses at lower BMIs. Similarly, individuals with high BMIs and detectable diethylphosphate had higher triglyceride than those without detectable levels, while minimal differences between diethylphosphate detection statuses were observed at lower BMIs (interaction: *P* = 0.02). Thus, cardiometabolic health outcome differs depending on the specific OP metabolite being examined, with higher BMIs amplifying health risk.

## 1. Introduction

Organophosphates (OP) are one of the most common types of pesticide used around the world [1] and individuals can come into contact with them through a variety of avenues such as ingestion of contaminated fruits and vegetables, contact with residential pest control applications or through their occupation. OP pesticides can enter the body through ingestion and inhalation, as well as direct contact with the skin [2, 3]. Once in the body, the liver processes the pesticide and its metabolites are excreted through the urine [3]. The rate of breakdown and thus sensitivity to pesticides vary between individuals and are related to differences in genetics and enzymatic activity [4]. The metabolites produced are unique for each pesticide, with the most commonly studied being dimethylphosphate (DMP), dimethylthiophosphate (DMTP), dimethyldithiophosphate (DMDTP), diethylphosphate (DEP), diethylthiophosphate (DETP), and diethyldithiophosphate (DEDTP) [5].

Previous literature has demonstrated OP pesticide exposure to be associated with elevated cardiometabolic risk such as increased triglycerides [6–9], HDL [7, 8], hyperglycemia [7, 10–14], and blood pressure [15–17]. In addition, positive associations between weight gain and OP pesticides have also been observed [13, 18–20]. However, the majority of studies were performed on animals or individuals with high occupational OP pesticide exposure and it is unclear whether lower levels of OP more commonly observed in the general population are also associated with adverse health effects. Furthermore, as obesity is associated with negative health outcomes, it is also unclear if the influence of OP pesticides on cardiometabolic health risk remains independent of BMI.

Therefore, the main objective of this study is to determine whether OP metabolites modify the relationship between cardiometabolic health risks and BMI in the general US population.

## 2. Research Design and Methods

### 2.1. National Health and Nutrition Examination Survey (NHANES)

NHANES is a national survey that aims to collect health and diet information from a representative sample of the noninstitutionalized US population. NHANES continuously (1999–2013) utilizes a multifaceted probability sampling design that places importance on the oversampling of minority populations. All participants provided written informed consent in agreement with the Public Health Service Act prior to any data collection. Information was acquired from participants through household questionnaires, telephone interviews, and examinations conducted by health care professionals and trained personnel. Data examined in this study was the public use microdata files accessed from the Center for Disease Control and Prevention (CDC) website [21]. Information on NHANES survey methods is described in greater detail elsewhere [22, 23].

### 2.2. Study Participants and Exclusion Criteria

A total of 51 623 participants were examined during the 1999–2008 survey years. Within this population, NHANES randomly selected a subsample of 12 273 survey participants for assessment of OP pesticide exposure using urinary concentrations of six types of dialkyl phosphate (DAP) metabolites. Individuals <20 years of age (*n* = 5452) or pregnant participants (*n* = 417) were excluded from the study resulting in 6467 participants. Individuals with a fasting duration of less than 3 hours or more than 24 hours (*n* = 1231) or those with missing or outlier measurements for high-density lipoprotein cholesterol (HDL; *n* = 296), low-density lipoprotein cholesterol (LDL; *n* = 3626), total cholesterol (*n* = 296), serum triglyceride (*n* = 3467), plasma glucose (*n* = 3443), serum insulin (*n* = 3483), glycohemoglobin (HbA1c; *n* = 243), homeostatic model assessment of insulin resistance (HOMA-IR; *n* = 3488), systolic blood pressure (SBP; *n* = 289), diastolic blood pressure (DBP; *n* = 322), C-reactive protein (CRP; *n* = 277), or body mass index (BMI; *n* = 120) were also excluded. In addition, missing and outlier values for urinary creatinine (*n* = 4), smoking status (*n* = 6), and PIR (*n* = 491) were excluded. One individual with an extreme outlier of 2800 *μ*g/L for DMTP was also excluded. For each variable, outliers were considered to be physiologically implausible values and notable points of influence that strengthened the association between health and BMI. Finally, the mean caloric intake was substituted for all individuals with missing caloric intake values (*n* = 278) resulting in 2227 participants available for analysis.

### 2.3. Assessment of Organophosphate Pesticide Exposure

A multistage approach was employed for the storage, transportation, and measurement of each OP metabolite. Urine specimens were collected, stored, and then placed over dry ice to be transported to the Division of Laboratory Sciences. Once at the lab, urine samples were brought to room temperature and spiked with stable isotope analogues of the specific DAP metabolite being measured (providing a reliable internal control). The remaining products were further processed to obtain DAP metabolite measurements [24]. These methods are described in greater detail elsewhere [1, 24].

Sufficient metabolite concentrations were required for the instruments to accurately detect the OP metabolites, with instrument sensitivity varying depending on survey year. Values below the detection limit were replaced with a value equal to the detection limit divided by the square root of two. For this study, the survey year with the highest detection limit was used as the cut-off value for each metabolite, dichotomizing participants into those above and below metabolite detection limit.

### 2.4. Cardiometabolic Risk Factors

Cardiometabolic risk factors were analyzed using a number of different techniques. In general, trained phlebotomists at mobile examination centers (MEC) obtained blood samples from survey participants. CRP was obtained through latex-enhanced nephelometry of blood specimens [25]. The hexokinase method and Roche/Hitachi analyzer were used to evaluate blood plasma glucose [26], while blood HbA1c and insulin levels were obtained by ion exchange high-performance liquid chromatography using the Primus apparatus [27] and the immunoassay method, respectively [28]. HOMA-IR was calculated by dividing the product of fasting plasma glucose (mmol/L) and insulin (mU/L) by 22.5. Hitachi analyzers were used to quantify triglyceride, total cholesterol, and HDL levels throughout the survey years while LDL was calculated using the Friedewald equation [29–31]. Blood pressure was measured 3 times with some individuals being evaluated 4 times in the event of equipment or technician error. For this study, the mean of all available DBP and SBP measurements was used during analysis. A more detailed explanation of the methods can be found online [21].

### 2.5. Statistical Analysis

Participant characteristics by OP metabolite detection limit status were examined using chi-square tests for categorical variables and *t*-tests for continuous variables. Continuous variables are presented as means ± standard error (SE) while the prevalence (N, %) was presented for categorical variables. Multiple regression analysis was performed to assess the association between OP metabolites and BMI. Each regression model was adjusted for potential confounders including age, sex, ethnicity, PIR, smoking status, fasting duration, total caloric intake, and urinary creatinine levels to account for urinary dilution level [22]. All regression models included an interaction term between BMI and OP metabolites. If no significant interaction was observed, the interaction term was excluded and main effects were examined with adjusted least square means (LSM) ± SE being computed to illustrate the differences in cardiometabolic health risk by OP metabolite detection status. All data was analyzed using SAS version 9.3 survey procedures including appropriate weights to adjust for unequal sampling probabilities to represent the US population. A value of *P* ≤ 0.05 was used as the criterion for significance.

## 3. Results

Participant characteristics by OP metabolite detection status are presented in Table 1. In general, those with detectable OP metabolites were significantly older than those below detection (with the exception of DETP and DEDTP). In addition, BMI was significantly (*P* < 0.0001) associated with all observed cardiometabolic health risk factors after adjustment for confounders.

Table 2 illustrates the association between OP metabolites and cardiometabolic health risks after adjusting for confounders. DBP was observed to be significantly higher for individuals with detectable DMP, DEP, or DEDTP (*P* < 0.05) while no significant difference was observed for DMTP, DMDTP, or DETP. Further, individuals with detectable DMTP had significantly lower total cholesterol and LDL than those without detectable levels, while no significant differences were observed for CRP, HOMA-IR, HbA1c, plasma glucose, or SBP (*P* > 0.05).

**Figure 1:**
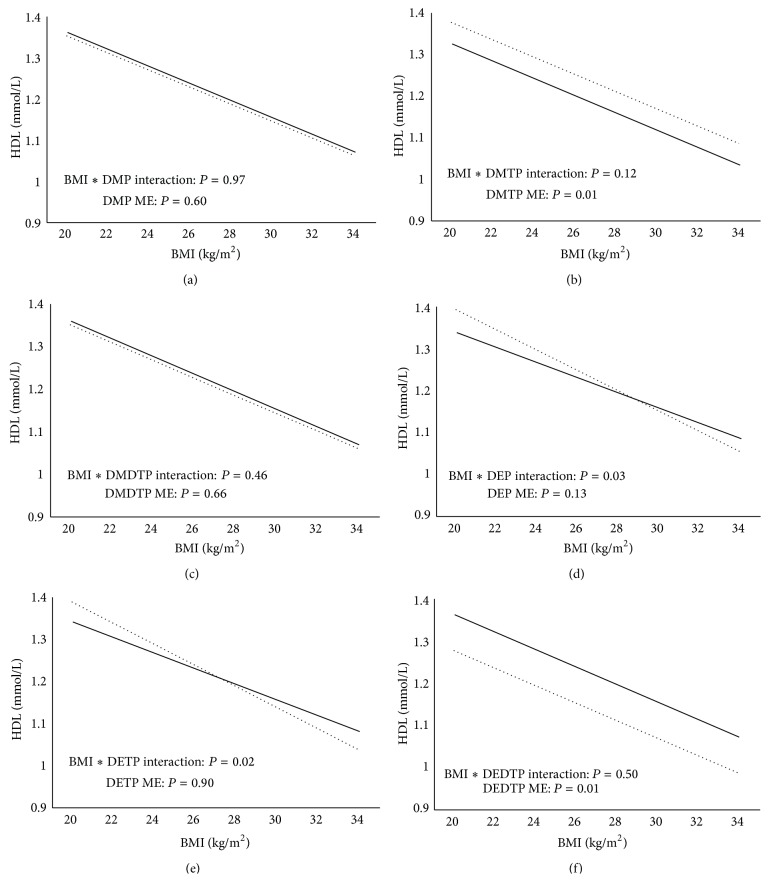
((a)–(f)) Relationship between HDL and BMI by DMP (a), DMTP (b), DMDTP (c), DEP (d), DETP (e), and DEDTP (f) detection limit status. Solid black line represents individuals below the detection limit while the dotted black line represents individuals above the detection limit. ME = main effect. Models are adjusted for PIR, ethnicity, age, sex, smoking status, urinary creatinine, fasting duration, and total calorie intake.

Figure 1 presents the association between HDL and BMI by OP metabolite detection limit status after adjustment for confounders. Individuals with detectable DMTP had significantly higher HDL than those below detectable levels (*P* = 0.01). Further, those with detectable DEDTP had significantly lower HDL levels than those below detection (*P* = 0.01). Individuals with detectable DEP and DETP exhibited lower HDL at the higher BMI range, while a marginal difference was observed between OP metabolite detection statuses at lower BMIs (interaction effect: *P* = 0.034 and *P* = 0.0153, resp.). No significant interactions or main effects were observed with DMP or DMDTP and HDL (*P* > 0.05).

**Figure 2:**
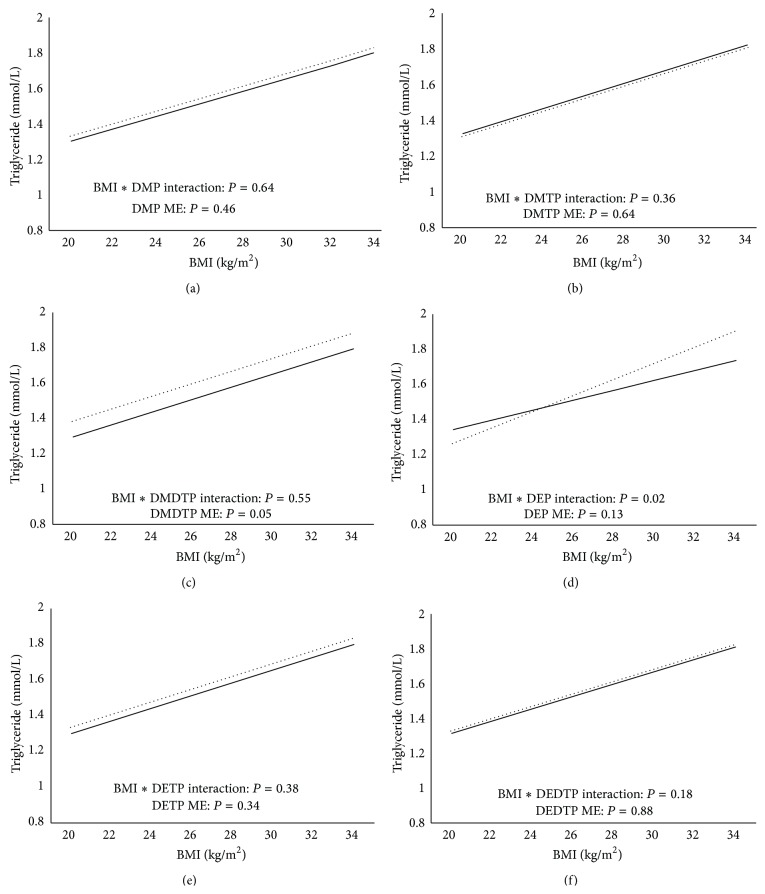
((a)–(f)) Relationship between triglyceride and BMI by DMP (a), DMTP (b), DMDTP (c), DEP (d), DETP (e), and DEDTP (f) detection limit status. Solid black line represents individuals below the detection limit while the dotted black line represents individuals above the detection limit. ME = main effect. Models are adjusted for PIR, ethnicity, age, sex, smoking status, urinary creatinine, fasting duration, and total calorie intake.

Figure 2 demonstrates the relationship between triglyceride and BMI by OP metabolite detection limit status after adjusting for confounders. Individuals with detectable DMDTP exhibited significantly higher triglyceride levels than those below detection (main effect: *P* = 0.05). In addition, there was a significant interaction effect between DETP and BMI (*P* = 0.02), wherein individuals with detectable DETP had higher triglyceride levels than those below detection at higher BMI ranges and only a minimal difference at lower BMIs. There were no significant interactions or main effects for DMP, DMTP, DEP, or DEDTP metabolites (*P* > 0.05).

**Figure 3:**
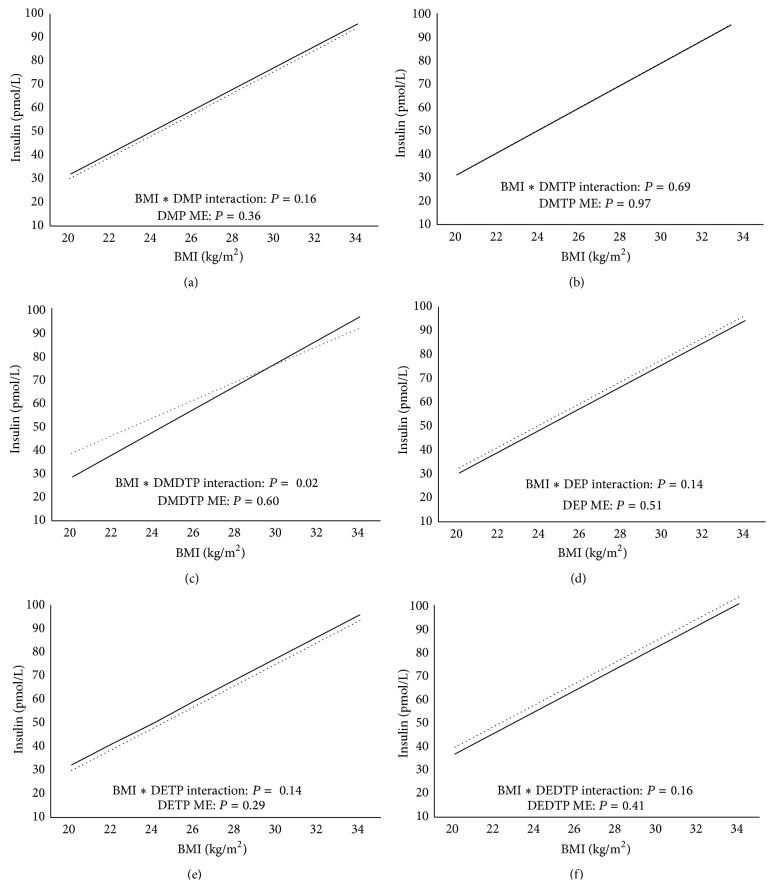
((a)–(f)) Relationship between insulin and BMI by DMP (a), DMTP (b), DMDTP (c), DEP (d), DETP (e), and DEDTP (f) detection limit status. Solid black line represents individuals below the detection limit while the dotted black line represents individuals above the detection limit. ME = main effect. Models are adjusted for PIR, ethnicity, age, sex, smoking status, urinary creatinine, fasting duration, and total calorie intake.

BMI was positively associated with insulin levels. Individuals with detectable DEP had higher insulin levels than those below detection at the higher BMI range with smaller differences at a lower BMI (Figure 3; *P* = 0.0191). No significant associations were observed with the other metabolites and insulin (*P* > 0.05).

## 4. Discussion

To our knowledge, we are one of the first to investigate the influence of urinary OP metabolites on the relationship between BMI and cardiometabolic health risk in the general population. We demonstrated that individuals with detectable OP metabolites most commonly have an augmentation of obesity-related cardiometabolic health risk. However, DMTP was associated with a healthier lipid profile for a given BMI. These findings suggest that detectable OP metabolite levels in the general population may confer both adverse and advantageous health outcomes, thus highlighting the importance of examining each metabolite individually when studying the effects of OP metabolites on health.

Several studies have demonstrated significant findings in respect to OP pesticide and glucose markers [7, 10–14, 19, 20, 32]. In general, it is believed that OP pesticide directly affects the functioning of the pancreas leading to an altered glucose profile [33]. Consequently, a large number of animal and human studies have observed OP pesticide exposure to be associated with significant increases in plasma glucose [7, 10, 11, 14, 32], insulin [32], insulin resistance [13, 32], and incidence of type 2 diabetes [12]. Although previous literature has established links between OP pesticide exposure and diabetes biomarkers, we only observed DMDTP to be associated with fasting insulin after adjusting for BMI. The lack of significance between glucose markers and OP metabolites may be partly due to the fact that previous investigation of human exposure to OP pesticides has focused primarily on individuals who are regularly exposed to high quantities of the pesticides through their occupation, such as farmers [11, 13] and pesticide applicators [12]. Thus, OP exposure levels seen in the broader population may not be sufficient to elicit the same negative effects.

The association between OP pesticide exposure and HDL remains unclear, with previous studies reporting a number of contradictory findings. Compared to controls, rats exposed to OP pesticides have exhibited significantly lower [7, 8], significantly higher [6, 9, 34], and no significant [35] effect on HDL. Mechanisms for such findings are unclear, but it is suggested that differences in enzymatic activity of paraoxonase-1 (PON1) may play a role in the rate of breakdown of OP pesticides [4, 36–38] and also influence HDL and LDL levels [39, 40]. OP pesticides may also influence lipase activity of hepatic triglycerides and plasma lipoproteins(8). We demonstrate that individuals with detectable DEDTP had 0.09 mmol/L lower HDL than those with nondetectable levels for a given BMI (*P* = 0.01). In addition, obese individuals above detection for DEP and DETP had 0.03–0.04 mmol/L lower HDL compared to those below detection. Based on previous observations [41], these findings may translate to 4–15% higher cardiovascular disease (CVD) mortality rates. However, why the association between obesity and HDL is modified differently by different OP pesticides is still unclear and warrants further investigation. Obesity itself is known to have several epigenetic and metabolic effects [42] and, from the results here, it is clear that, for certain OP pesticides, there is a differential effect that is depending on the level of obesity of the individual. This is akin to pharmacotherapy dosing differences in adults with obesity that extends beyond simple differences in weight and body surface area [43]. Thus, research is needed to understand how these physiological differences associated with obesity may interact with the complex physiological changes associated with OP pesticides and the resultant health effects.

Although having detectable levels of certain metabolites was associated with a detrimental health profile, the opposite finding was observed with the DMTP metabolite. In general, individuals with detectable DMTP exhibited significantly higher HDL, lower LDL, and lower total cholesterol than those without detectable levels. Interestingly, these findings are not completely uncommon with previous reports demonstrating that OP pesticide exposure decreases LDL in normolipidemic and hyperlipidemic rats [34] and rabbits [9]. As previously discussed, a number of studies have also observed higher HDL levels [6, 9, 34] in OP pesticide exposed participants. Although interesting, it remains unclear why this specific metabolite resulted in a more favorable lipid outcome; however, we believe that these findings place emphasis on the unique physiological effects of each specific OP metabolite. Thus, we are in need of further research examining the possible etiology and physiology of DMTP within the body to advance our understanding of these outcomes.

Previous work on rodents has examined the relationship between OP pesticide exposure and blood lipids with reports in which OP pesticide exposed rats exhibit significantly higher triglyceride levels than controls [6–9]. Consistent with these studies on rats, we demonstrate that individuals with detectable DMDTP have 0.09 mmol/L higher serum triglyceride levels than those below detection, independent of BMI (*P* = 0.05). Further, obese individuals with detectable DEP had 0.17 mmol/L higher triglyceride than those below detection which translates to a 5–13% higher risk of CVD [44]. Thus, certain OP metabolites foster unfavorable triglyceride levels, which may modestly augment CVD in the general population, with individuals at a higher BMI range having their risk amplified the most.

Limited research has examined the effects of OP pesticides on blood pressure; however it has been reported that both low and high doses of OP pesticide are associated with an increase in blood pressure up to 24 hours after exposure in rats [15]. In addition, rats with preexisting hypertension exhibit greater blood pressure increases after the administration of the OP pesticide [17]. These findings are thought to be the result of OP pesticides directly affecting the central and peripheral nervous system pathways, leading to altered blood pressure outcomes [15]. Interestingly, we observed that, independent of BMI, individuals with detectable DMP, DEP, and DEDTP metabolite levels had a 1–3 mmHg higher DBP (*P* < 0.05) than those below detection which may augment the risk of CHD by 4–13% [45]. Therefore, independent of BMI, the association between OP metabolites and blood pressure may amplify risk of adverse cardiac events in the general population.

A few limitations and strengths of this study warrant mention. First, the cross-sectional nature of the present study does not allow us to infer causation. Second, although several previous studies support the use of these six types of dialkyl phosphate metabolites as an indication of OP pesticide exposure [9, 46, 47], questions on the ability to attribute the source of urinary OP metabolites remain [48, 49]. Further, individual differences in the metabolism of and sensitivity towards OP pesticides due to variations in PON1 polymorphisms and enzymatic efficiency [4, 36, 38] may have also influenced our results. Finally, we elected to use more liberal detection limits to reduce the likelihood of misclassifying the exposure, and this study represents one of the few studies examining OP metabolite concentrations and health risk that is more applicable to the general public.

## 5. Conclusions

In summary, we demonstrated that having detectable levels of urinary OP metabolites might be associated with both advantageous and detrimental metabolic outcomes independent of BMI, with obese individuals having an amplification of cardiometabolic health risk. In addition, when studying the effects of OP metabolites on health, it is important to examine the influence of each OP metabolite individually as their effects on health may differ. Consequently, future studies need to examine the physiology of each metabolite, with particular attention on the obese population wherein health risk seems to be amplified.

## Figures and Tables

**(a):** 

	DMP			DMTP			DMDTP		
	Below (*n* = 1299)	Above (*n* = 928)	*P* value	Below (*n* = 740)	Above (*n* = 1487)	*P* value	Below (*n* = 1636)	Above (*n* = 591)	*P* value
Age (years)	43.5 ± 0.5	45.4 ± 0.6	<0.01	42.5 ± 0.7	45.3 ± 0.5	<0.0001	43.7 ± 0.4	45.8 ± 0.7	<0.01
Sex, *n* (% male)	704 (54.2)	452 (48.7)	0.04	386 (52.2)	770 (51.8)	0.25	872 (53.3)	284 (48.1)	0.03
BMI (kg/m^2^)	28.1 ± 0.2	27.9 ± 0.3	0.44	27.6 ± 0.2	28.2 ± 0.2	0.01	28.0 ± 0.2	28.0 ± 0.3	0.90
Metabolic variables									
HOMA-IR	2.92 ± 0.09	2.74 ± 0.09	0.10	2.68 ± 0.10	2.93 ± 0.08	0.03	2.84 ± 0.08	2.84 ± 0.12	0.98
Insulin (pmol/L)	67.4 ± 1.9	64.2 ± 1.8	0.15	63.9 ± 2.0	67.2 ± 1.7	0.16	65.8 ± 1.5	66.7 ± 2.8	0.71
HbA1c (%)	5.4 ± 0.1	5.4 ± 0.1	0.86	5.4 ± 0.1	5.5 ± 0.1	0.03	5.4 ± 0.1	5.5 ± 0.1	0.33
Glucose (mmol/L)	5.60 ± 0.04	5.58 ± 0.04	0.68	5.51 ± 0.05	5.63 ± 0.04	0.04	5.59 ± 0.03	5.58 ± 0.06	0.79
HDL (mmol/L)	1.35 ± 0.01	1.37 ± 0.02	0.33	1.33 ± 0.02	1.37 ± 0.02	0.03	1.35 ± 0.01	1.37 ± 0.02	0.39
LDL (mmol/L)	3.11 ± 0.03	3.07 ± 0.03	0.31	3.21 ± 0.04	3.03 ± 0.03	<0.0001	3.11 ± 0.03	3.06 ± 0.05	0.30
Total cholesterol (mmol/L)	5.13 ± 0.04	5.11 ± 0.04	0.65	5.21 ± 0.04	5.07 ± 0.03	<0.01	5.12 ± 0.03	5.13 ± 0.06	0.85
Triglyceride (mmol/L)	1.47 ± 0.03	1.47 ± 0.03	0.56	1.45 ± 0.04	1.47 ± 0.02	0.56	1.44 ± 0.02	1.52 ± 0.04	0.03
CRP (nmol/L)	34.3 ± 1.9	37.1 ± 2.9	0.15	34.3 ± 2.9	36.2 ± 1.0	0.48	35.2 ± 1.9	35.2 ± 2.9	0.85
SBP (mmHg)	120 ± 1	120 ± 1	0.93	119 ± 1	121 ± 1	0.06	120 ± 1	120 ± 1	0.93
DBP (mmHg)	71 ± 1	72 ± 1	<0.01	72 ± 1	72 ± 1	0.87	71 ± 1	72 ± 1	0.29

DMP = dimethylphosphate, DMTP = dimethyldiphosphate, and DMDTP = dimethyldithiophosphate.

Values are presented as mean ± SE.

*P* values represent the statistical difference between detection statuses.

**(b):** 

	DEP			DETP			DEDTP		
	Below (*n* = 1282)	Above (*n* = 945)	*P* value	Below (*n* = 1376)	Above (*n* = 851)	*P* value	Below (*n* = 2109)	Above (*n* = 118)	*P* value
Age (years)	43.6 ± 0.6	45.1 ± 0.6	0.02	44.7 ± 0.4	43.6 ± 0.6	0.11	44.5 ± 0.4	40.6 ± 1.4	<0.01
Sex *n* (% male)	693 (54.1)	463 (49.0)	0.05	701 (50.9)	455 (53.4)	0.10	1098 (52.1)	58 (49.2)	0.30
BMI (kg/m^2^)	28.0 ± 0.2	27.9 ± 0.2	0.53	28.2 ± 0.2	27.7 ± 0.2	0.06	28.0 ± 0.2	27.6 ± 0.6	0.42
Metabolic variables									
HOMA-IR	2.82 ± 0.09	2.87 ± 0.13	0.63	2.90 ± 0.09	2.74 ± 0.09	0.17	2.85 ± 0.07	2.80 ± 0.21	0.86
Insulin (pmol/L)	65.5 ± 2.0	66.7 ± 2.6	0.60	67.2 ± 1.7	64.1 ± 1.8	0.19	66.0 ± 1.4	67.2 ± 4.7	0.79
HbA1c (%)	5.4 ± 0.1	5.4 ± 0.1	0.55	5.5 ± 0.1	5.4 ± 0.1	0.10	5.4 ± 0.1	5.3 ± 0.1	0.01
Glucose (mmol/L)	5.59 ± 0.05	5.59 ± 0.04	0.95	5.61 ± 0.04	5.56 ± 0.04	0.40	5.60 ± 0.03	5.40 ± 0.07	0.09
HDL (mmol/L)	1.35 ± 0.02	1.37 ± 0.02	0.22	1.36 ± 0.01	1.35 ± 0.02	0.57	1.36 ± 0.01	1.28 ± 0.04	0.02
LDL (mmol/L)	3.08 ± 0.03	3.12 ± 0.04	0.35	3.12 ± 0.03	3.05 ± 0.04	0.08	3.10 ± 0.02	3.05 ± 0.11	0.55
Total cholesterol (mmol/L)	5.09 ± 0.03	5.17 ± 0.04	0.07	5.15 ± 0.03	5.07 ± 0.04	0.09	5.13 ± 0.03	4.98 ± 0.15	0.11
Triglyceride (mmol/L)	1.44 ± 0.03	1.50 ± 0.03	0.07	1.46 ± 0.02	1.46 ± 0.03	0.97	1.47 ± 0.02	1.43 ± 0.10	0.65
CRP (nmol/L)	34.3 ± 1.0	36.2 ± 2.9	0.37	37.1 ± 1.9	32.4 ± 1.9	0.03	35.2 ± 1.0	35.2 ± 7.6	0.99
SBP (mmHg)	120 ± 1	121 ± 1	0.08	121 ± 1	119 ± 1	0.07	120 ± 1	119 ± 2	0.38
DBP (mmHg)	71 ± 1	72 ± 1	<0.01	72 ± 1	72 ± 1	0.90	71 ± 1	73 ± 1	0.04

DEP = diethylphosphate, DETP = diethyldiphosphate, and DEDTP = diethyldithiophosphate.

Values are presented as mean ± SE.

*P* values represent the statistical difference between detection statuses.

**Table 2:** Cardiometabolic risk factors means for individuals above and below OP metabolite detection limit.

OP metabolite	LDL (mmol/L)	Total cholesterol (mmol/L)	Glucose (mmol/L)	HOMA-IR	HbA1c (%)	CRP (nmol/L)	SBP (mmHg)	DBP (mmHg)
DMP								
Below	3.06 ± 0.08	5.08 ± 0.08	5.78 ± 0.09	3.11 ± 0.18	5.6 ± 0.1	39.1 ± 3.8	122 ± 1	71 ± 1^*∗*^
Above	3.04 ± 0.09	5.05 ± 0.10	5.74 ± 0.09	2.96 ± 0.15	5.6 ± 0.1	42.9 ± 5.7	122 ± 1	72 ± 1
DMTP								
Below	3.18 ± 0.09^*∗*^	5.17 ± 0.09^*∗*^	5.75 ± 0.08	3.01 ± 0.15	5.6 ± 0.1	40.0 ± 5.7	122 ± 1	72 ± 1
Above	2.98 ± 0.08	5.01 ± 0.09	5.77 ± 0.09	3.07 ± 0.17	5.6 ± 0.1	41.0 ± 4.8	122 ± 1	71 ± 1
DMDTP								
Below	3.06 ± 0.08	5.07 ± 0.09	5.78 ± 0.09	3.05 ± 0.16	5.6 ± 0.1	41.0 ± 4.8	122 ± 1	71 ± 1
Above	3.02 ± 0.09	5.06 ± 0.10	5.73 ± 0.10	3.04 ± 0.18	5.6 ± 0.1	40.0 ± 4.8	122 ± 1	72 ± 1
DEP								
Below	3.04 ± 0.08	5.04 ± 0.09	5.77 ± 0.09	3.01 ± 0.17	5.6 ± 0.1	40.0 ± 4.8	121 ± 1	71 ± 1^*∗*^
Above	3.07 ± 0.08	5.10 ± 0.09	5.76 ± 0.09	3.10 ± 0.19	5.6 ± 0.1	41.0 ± 5.7	122 ± 1	72 ± 1
DETP								
Below	3.07 ± 0.08	5.07 ± 0.09	5.78 ± 0.08	3.10 ± 0.16	5.6 ± 0.1	41.9 ± 4.8	122 ± 1	71 ± 1
Above	3.03 ± 0.09	5.05 ± 0.10	5.74 ± 0.10	2.97 ± 0.18	5.6 ± 0.1	39.1 ± 4.8	121 ± 1	71 ± 1
DEDTP								
Below	3.05 ± 0.08	5.07 ± 0.09	5.77 ± 0.09	3.04 ± 0.16	5.6 ± 0.1	41.0 ± 4.8	122 ± 1	71 ± 1^*∗*^
Above	3.05 ± 0.12	4.99 ± 0.15	5.67 ± 0.10	3.13 ± 0.21	5.5 ± 0.1	40.0 ± 8.6	123 ± 2	74 ± 1

^*∗*^Significant difference between detection statuses (*P* < 0.05).

Values are means ± SE adjusted for BMI, PIR, ethnicity, age, sex, smoking status, urinary creatinine level, fasting duration, and total

caloric intake.

DMP = dimethylphosphate, DMTP = dimethylthiophosphate, DMDTP = dimethyldithiophosphate, DEP = diethylphosphate,

DETP = diethylthiophosphate, and DEDTP = diethyldithiophosphate.
